# Cardiothoracic 3D Printing: Workflow and Applications for Clinical Practice

**DOI:** 10.1186/1749-8090-10-S1-A62

**Published:** 2015-12-16

**Authors:** Thomas Foley, Rakesh Suri, Shuai Leng, Jonathan Morris, Jane Matsumoto, Eric Williamson

**Affiliations:** 1 Radiology, Mayo Clinic, Rochester, MN, USA

## Background/Introduction

Recent advances in anatomic rendering software have improved our ability to convert image datasets into file formats that can be used to create highly detailed cardiothoracic models. Parallel improvements in 3D printer technology have decreased the time necessary to produce physical representations of these models. We will describe the use of these models to assist with our cardiothoracic surgical practice.

## Aims/Objectives

1. Describe how highly detailed radiographic imaging datasets are transformed into useful life-sized models through 3D printing.

2. Discuss how these models aid in planning of open surgical and minimally invasive procedures across a wide range of cardiothoracic diseases.

## Method

We retrospectively reviewed our database of 3D printed anatomic models and catalogued the cases based on anatomic region. This list was cross-referenced to determine which patients underwent a cardiothoracic surgical procedure. Surgical reports were reviewed and, when appropriate, outcomes were discussed with the surgical team to determine how the 3D models were employed to optimize patient care.

## Results

Since the adoption of 3D printing in our practice, we have created well over 100 anatomic models for use in clinical practice, as well as research and education. A large subset of these models have been used to plan surgical and minimally invasive cardiothoracic procedures. In this exhibit, we will describe the equipment and workflow used in our practice to: 1) acquire and import high resolution imaging datasets, 2) segment the imaging data to produce an anatomically accurate model, and 3) create a highly detailed physical model of the relevant anatomy. Additionally, we will use actual case examples to demonstrate how these models have been used to improve safety, efficiency and outcomes of cardiothoracic procedures.

## Discussion/Conclusion

Anatomic models produced using 3D printing techniques provide an accurate depiction of relevant anatomy that can assist in planning of open and minimally invasive surgical procedures in a wide range of cardiothoracic diseases.

